# Trombiculiasis in 4 Dogs with Neurologic Signs, the Netherlands, 2024

**DOI:** 10.3201/eid3112.241758

**Published:** 2025-12

**Authors:** Koen M. Santifort, Hannah A. Reijmerink

**Affiliations:** Evidensia Small Animal Hospital, Arnhem, the Netherlands (K.M. Santifort); Evidensia Small Animal Hospital Hart van Brabant, Waalwijk, the Netherlands (K.M. Santifort); Dierenartspraktijk Edam, Edam, the Netherlands (H.A. Reijmerink)

**Keywords:** Trombiculiasis, *Neotrombicula autumnalis*, canine, mites, parasites, ataxia, infestation, harvest mite, the Netherlands

## Abstract

Sporadic cases of trombiculiasis have been reported as causing neurologic signs in dogs. We report a cluster of trombiculiasis cases (outbreak) in the province of Noord-Holland, the Netherlands, associated with *Neotrombicula autumnalis* mite infestation. Veterinarians should consider *N. autumnalis* infestation in differential diagnoses for acute onset neurologic signs in dogs.

Neurotoxicologic disorders in dogs can be associated with parasites, of which tick bite paralysis is an example (*1*–*3*). Ticks commonly associated with acute flaccid paralysis in animals are *Dermacentor variabilis*, *D. andersoni*, and *Ixodes holocyclus* (*1*–*3*). In the Netherlands, parasite-associated neurotoxicologic syndromes are not commonly included in differential diagnoses lists. However, members of the mite family *Trombiculidae* (chigger or harvest mites) are represented in the Netherlands, among them *Neotrombicula autumnalis*. *N. autumnalis* larvae are ≈0.2 mm in length, bright orange, with 6 legs, a rectangular scutum bearing 3 pairs of setae, and 5-segmented palps with a characteristic trifurcate seta on the third segment. Trombiculiasis (trombiculosis, trombidiosis) is the skin condition most associated with larvae infestation of this mite family. Researchers have reported sporadic cases of dogs with neurologic signs concurrent with trombiculiasis (*4*,*5*). We document a cluster of dogs whose owners sought veterinary care for observed neurologic signs presumptively associated with *N. autumnalis* infestations.

In August 2024, owners sought care for a 9.5-year-old male Finnish Lapphund at a veterinary clinic near Edam, the Netherlands (Noord-Holland province). The dog demonstrated acute progressive signs, including vomiting, hyperpnea, licking and biting between toes (itch), salivation, hyperthermia (39.5°C; normal 38°C–39°C), and generalized ataxia and paresis. Clinicians observed red-orange mites, morphologically identified as *N. autumnalis*, on the skin of the paws and abdomen and concurrent erythema. An injection of dexamethasone administered 1 day prior to the dog’s arrival at the clinic relieved some signs (itch), but owners observed progressing neurologic signs. The dog was alert and responsive, but no longer ambulatory 12 hours after onset; attempts at rising were unsuccessful. Because of the severity of the neurologic signs, the owners elected euthanasia.

In the same week, 3 more dogs arrived at a veterinary clinic in the Edam area. Those dogs showed acute progressive signs, including generalized proprioceptive ataxia and paresis; itch, predominantly of the interdigital regions of all 4 paws; interdigital erythema; hyperthermia; vomiting; and hyperpnea. Clinicians noted *N. autumnalis* aggregations on the skin of the paws and abdomen. In 2 cases, dog owners reported seeing red-orange stains between digits, which upon closer examination were identified as large accumulations of mites. During a brief period, all dogs were nonambulatory. Details provided by 1 of the dog owners included an acute onset of ataxia and paresis, most notably in the pelvic limbs (Video), which progressed over 48 hours to a nonambulatory state. Thereafter, that dog showed progressive recovery of neurologic function.

**Video:** Footage obtained from a study of trombiculiasis in 4 dogs with neurologic signs, the Netherlands, 2024. Video shows proprioceptive ataxia and paresis of all 4 limbs in a rough collie dog (7-year-old male) infested with *Neotrombicula autumnalis* mites.

All 3 dogs received treatment, including injections with corticosteroids, application of detergents to the paws, and topical ectoparasiticides. Both nonambulatory dogs recovered ambulation within 12 hours. In 1 of those 2 dogs, clinicians noted signs of neurologic improvement before initiating antiparasitic treatment. None of the dogs required continued medical treatment.

Interviews with owners revealed that all 4 dogs had been walked in a seaside area with dunes between Edam and Warder, Noord-Holland, the Netherlands (coordinates 5232.0659 N 00503.3708 E). After veterinarians reported the cases to local authorities, officials posted warning signs at the entrance to the area, and local and social media reported the occurrence and disseminated the warning to the community. No other cases were forthcoming thereafter.

Ascertaining the likelihood of the mite infestations as the cause for clinical signs involved careful examination of both the clinical records and the literature (Appendix). Two previous reports documented the occurrence of neurologic signs in dogs from Austria and Spain with trombiculiasis (*4*,*5*). Another report documented severe signs of the condition, including weakness, loss of consciousness, vomiting, pain, and respiratory symptoms (1 case), but with no observed neurologic signs (*6*). *Neotrombicula inopinata* was the mite involved in the dogs affected in Spain (*5*). In the report from Austria, *N. autumnalis* was the infesting mite. Late summer and autumn (August–October) are risk periods mirrored in all reported cases.

In the previously reported cases of dogs in Austria with neurologic signs caused by *N. autumnalis*, authors described severe infestations of mites as follows: In dogs, when infestation is severe, the mites are so densely packed […] they resemble orange-red, brick-dust-like coatings or crusts. They often form clusters between the toes (translated from German) (*4*). That description mirrors our observation of orange-red staining on or between the digits. The clinical signs (e.g., vomiting) and neurologic signs reported in previous cases also closely resembled those observed in our cases (*4*,*5*). Paresis of the lower jaw also was reported in 2 dogs (*4*), a feature not seen in our cases.

In conclusion, *N. autumnalis* infestation should be considered a differential diagnosis for acute onset of neurologic signs in dogs that occur in late summer or autumn, especially in the presence of orange-red stains (on closer examination identified to be mites) on the digits, fur, or skin. Suspected cases warrant a careful inspection for mites to rule out infestation.

Appendix Additional information for trombiculiasis in 4 dogs with neurologic signs, the Netherlands, 2024.
