# Supplementary figures and images for: Trombiculiasis in 4 Dogs with Neurologic Signs, the Netherlands, 2024
Source: Emerg Infect Dis. 2025 Dec;31(12):2328–9. doi: 10.3201/eid3112.241758 (PMC12782184; doi:10.3201/eid3112.241758)

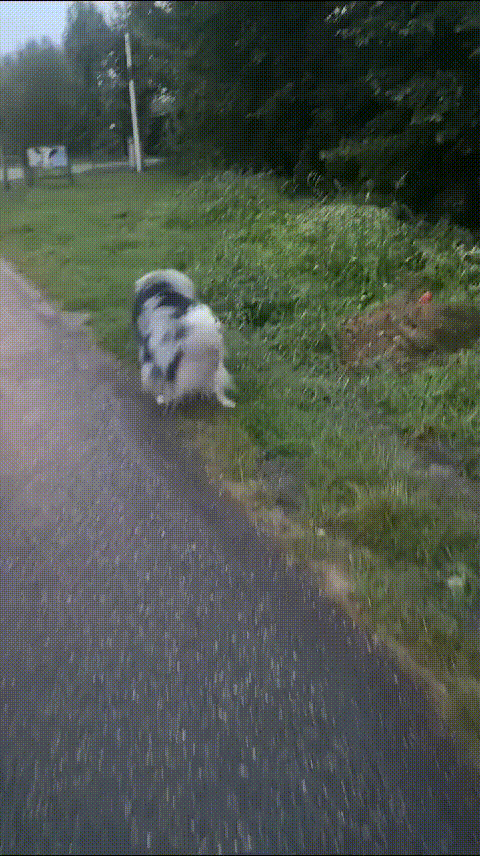

Supplement: Supplementary file 1 [file 24-1758-V.gif]
