# Supplementary material for: Trombiculiasis in 4 Dogs with Neurologic Signs, the Netherlands, 2024
Source: Emerg Infect Dis. 2025 Dec;31(12):2328–9. doi: 10.3201/eid3112.241758 (PMC12782184; doi:10.3201/eid3112.241758)
Supplement: Appendix — Additional information for trombiculiasis in 4 dogs with neurologic signs, the Netherlands, 2024. [file 24-1758-Techapp-s1.pdf]

Article DOI: http://doi.org/10.3201/eid3112.241758

EID cannot ensure accessibility for supplementary materials supplied by authors. Readers who have difficulty accessing supplementary content should contact the authors for assistance.

Trombiculiasis in 4 Dogs with Neurologic Signs, the Netherlands, 2024

Appendix

Prevention and treatment

Prevention of trombiculiasis might be achieved with the use of preventative ectoparasiticide medications (e.g., systemically active isoxazolines). Patient records did not include information on the use of such drugs before infestation with mites in the affected dogs reported here. We thus cannot conclude if the clinical signs could have been prevented by the use of such drugs. Treatment of trombiculiasis in dogs usually involves the use of ectoparasiticides. Fipronil, among others, has been reported to be effective in most instances (*1*). Repeated treatments may be necessary. In one of our reported cases, neurological signs already seemed to be improving before fipronil application. From the previous two reports, improvement was only seen after treatments were initiated (*2*,*3*). As the pathophysiology of the neurological signs is yet unknown (*3*), future observations may provide more answers to this question. If a neurotoxin is indeed excreted along with enzymes in the saliva of these mites, progressive neurological deterioration might be expected when mite infestations are not treated. If some auto-immune response or allergic response to components in the saliva of mites is at play, treatment with corticosteroids (included in treatment of all reported canine cases so far) or simply the passage of time itself may mitigate the neurological signs.

Diagnosis and zoonotic aspects

We cannot definitively exclude exposure to other neurotoxins or even other causes in general for neurological signs in the cases we describe in our report. However, the similarities in both the general signs (e.g., vomiting, dermatological signs) and neurological signs, the infestation with *N. autumnalis*, and temporal and geographic clustering of the cases is all highly

supportive of a causative role for the infestation with mites. The acute onset of neurological signs followed by a swift recovery without the need for continued medical treatment also fits with a neurotoxicologic etiology. Moreover, after local authorities took measures to warn owners not to walk dogs in the area where dogs were presumed to have been exposed to the mites, no more cases arose.

Importantly, a previous report mentions that owners of affected dogs as well as veterinarians treating the dogs had dermatologic signs and itch due to mite exposure either by concurrent infestation or transfer of mites from dogs to the persons (*2*). We do not have evidence of such signs in owners of our reported cases nor in their treating veterinarians. Trombiculiasis was reported to be a common cause of dermatologic signs in humans (pruritus, skin rash, papules) by a local health care practitioner in the Edam area (personal communication).

References

1. Lecru LA, Combarros D, Castilla-Castaño E, Navarro C, Cadiergues MC. Treatment of harvest mite infestation in dogs using a permethrin 54.5% and fipronil 6.1% (effitix®) topical spot-on formulation. Vet Sci. 2019;6:100. PubMed https://doi.org/10.3390/vetsci6040100

2. Prosl H, Rabitsch A, Brabenetz J. On the importance of the autumn grass mite–*Neotrombicula autumnalis* (Shaw 1790)–in veterinary medicine: nervous symptoms in dogs after massive infestation (*Trombiculid mite–Neotrombicula autumnalis* (Shaw 1790)–in veterinary medicine. Vet Pract. 1985;13:57–64.

3. Santibáñez P, Gallo E, Palomar AM, Portillo A, Carrillo JA, Oteo JA. Trombiculiasis in a dog with severe neurologic disorders, Spain. Emerg Infect Dis. 2020;26:819–20. PubMed https://doi.org/10.3201/eid2604.191313
